# Spatial and temporal imaging of long-range charge transport in perovskite thin films by ultrafast microscopy

**DOI:** 10.1038/ncomms8471

**Published:** 2015-06-23

**Authors:** Zhi Guo, Joseph S. Manser, Yan Wan, Prashant V. Kamat, Libai Huang

**Affiliations:** 1 Radiation Laboratory, University of Notre Dame, Notre Dame, Indiana 46556, USA; 2 Department of Chemical and Biomolecular Engineering, University of Notre Dame, Notre Dame, Indiana 46556, USA; 3 Department of Chemistry, Purdue University, West Lafayette, Indiana 47907, USA; 4 Department of Chemistry and Biochemistry, University of Notre Dame, Notre Dame, Indiana 46556, USA

## Abstract

Charge carrier diffusion coefficient and length are important physical parameters for semiconducting materials. Long-range carrier diffusion in perovskite thin films has led to remarkable solar cell efficiencies; however, spatial and temporal mechanisms of charge transport remain unclear. Here we present a direct measurement of carrier transport in space and in time by mapping carrier density with simultaneous ultrafast time resolution and ∼50-nm spatial precision in perovskite thin films using transient absorption microscopy. These results directly visualize long-range carrier transport of ∼220 nm in 2 ns for solution-processed polycrystalline CH_3_NH_3_PbI_3_ thin films. Variations of the carrier diffusion coefficient at the μm length scale have been observed with values ranging between 0.05 and 0.08 cm^2^ s^−1^. The spatially and temporally resolved measurements reported here underscore the importance of the local morphology and establish an important first step towards discerning the underlying transport properties of perovskite materials.

## Abstract (web summary)

Determining the mechanism of charge carrier transport in solar cells is important for their development towards higher efficiencies. Here, the authors elucidate the spatial and temporal diffusion of charge carriers in hybrid perovskite thin films through ultrafast transient absorption microscopy.

## Graphical abstract

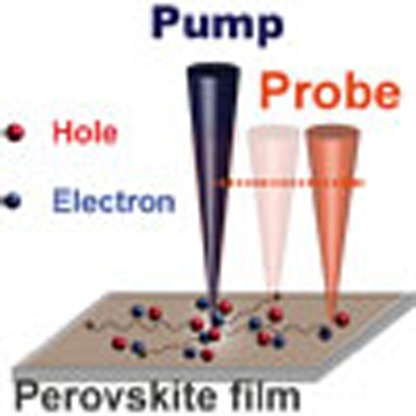

Charge transport in semiconductors is a crucial process that defines efficiency for optoelectronic devices such as solar cells and light-emitting diodes. Semiconducting organic–inorganic metal halide perovskites have recently been the focus of intense research, motivated largely by the rapid rise in efficiency of next-generation solar cells based on these materials. Development of perovskite solar cells over the past 5 years has yielded certified efficiencies as high as 20.1%, surpassing the performance of long standing dye sensitized, organic and amorphous silicon photovoltaic technologies^1^,^2^,^3^,^4^,^5^,^6^,^7^,^8^,^9^,^10^. A wide range of other applications utilizing lead halide perovskites such as photo- and electrocatalytic water splitting assemblies, optically pumped lasers, and light-emitting diodes have also been recently demonstrated^11^,^12^,^13^,^14^.

The exceptional characteristics of hybrid perovskites have brought their underlying optoelectronic properties under heavy scrutiny, including the nature of photogenerated species and the excited-state lifetime and decay pathways^15^,^16^,^17^,^18^. Of the various parameters that dictate perovskite device performance, the carrier diffusion length is particularly critical as it provides a benchmark for optimizing device thickness and morphology. The success of perovskite solar cells has in part been attributed to long carrier lifetimes and long-range diffusion on the order of 1 μm for both electrons and holes^16^,^18^,^19^,^20^,^21^,^22^,^23^,^24^. To-date, spectroscopic techniques such as photoluminescence (PL) quenching, transient terahertz photoconductance and time-resolved microwave conductivity have primarily been utilized to indirectly estimate carrier diffusion length (*L*) in lead halide perovskites^16^,^18^,^19^,^25^,^26^,^27^. However, results of these experiments have been incongruent, with determined *L* values varying more than an order of magnitude (100 nm–5 μm) in prototypical methylammonium lead iodide (CH_3_NH_3_PbI_3_) polycrystalline films. This can in part be attributed to the different excited-state processes (radiative versus non-radiative recombination) probed by the various techniques^26^. Most recently, carrier diffusion length of >175 μm has been measured in single crystals of lead halide perovskites, which establishes the upper limit for carrier transport in these materials^28^,^29^.

These divergent results are further convoluted by previous studies that report longer electron and hole diffusion lengths for the mixed halide perovskite CH_3_NH_3_PbI_3-*x*_Cl_*x*_ relative to its triiodide analogue^16^. Notably, however, chlorine has yet to be detected in the final mixed halide perovskite film^30^,^31^, suggesting that any discrepancies in electronic properties may stem from morphological rather than compositional differences. In light of these findings, it is evident that unravelling charge dynamics and transport mechanisms is complicated by the complex and heterogeneous nature of hybrid perovskite thin films, which strongly depends on processing conditions and precursors^30^,^32^. The use of bulk or ensemble spectroscopic techniques hinders reliable determination of the underlying material properties in hybrid perovskites.

Addressing these challenges requires spectroscopic tools with high spatial resolution that are capable of mapping charge dynamics and transport at the submicron scale. This can be achieved by combining microscopy techniques with ultrafast spectroscopic methods^33^,^34^,^35^. Prior work using ultrafast absorption microscopy has been used to study deactivation process of CH_3_NH_3_PbI_3_ perovskite film^36^. Probing carrier transport at the local rather than ensemble level can circumvent many of the inconsistencies that arise from perovskite sample preparation. Because carrier scattering and relaxation processes are often on the sub-nanosecond time scale, ultrafast time resolution is also necessary for elucidating these mechanisms.

Here we present direct measurement of carrier transport in both space and time by mapping carrier density in CH_3_NH_3_PbI_3_ with simultaneous femtosecond time resolution and ∼50-nm spatial precision using transient absorption microscopy (TAM). Using this technique, long-range carrier transport has been directly visualized in solution-processed CH_3_NH_3_PbI_3_ thin films. We successfully model spatial and temporal dependence of charge density using a diffusion model that includes higher order carrier recombination processes. Our measurements of charge carriers in polycrystalline CH_3_NH_3_PbI_3_ thin films yield diffusion constants that is two- to eightfold higher than those inferred from previous bulk PL-quenching measurements, pointing towards the importance of local morphology in controlling charge transport in the polycrystalline thin films.

## Results

### Structural and optical characterizations

**Figure 1:**
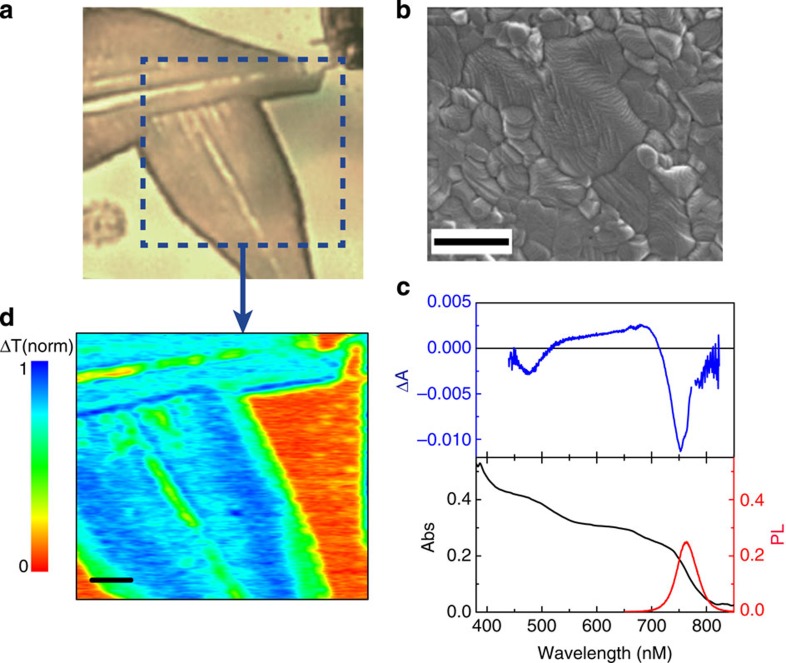
Structural and optical characterizations of CH_3_NH_3_PbI_3_ perovskite film. (**a**) Optical micrograph of a microcrystalline region in a CH_3_NH_3_PbI_3_ perovskite film. (**b**) Scanning electron micrograph (SEM) centred within a single representative perovskite flake. The scale bar is 0.5 μm. (**c**) Upper panel: transient absorbance spectrum measured at 50 ps in the bulk phase; lower panel: absorption and PL spectra of the sample region in (**a**). (**d**) Transient absorption imaging of the selected region of perovskite flake (dashed lines in **a**) at 0 ps. The whole image is 10 × 10 μm in size, with a scale bar of 1 μm.

An optical micrograph of the specific area used for TAM measurements reveals crystalline CH_3_NH_3_PbI_3_ islands on glass substrate (Fig. 1a). X-ray diffraction pattern of the sample shows sharp and strong perovskite (110) and (220) peaks, indicating that CH_3_NH_3_PbI_3_ film is crystalline (Supplementary Fig. 1). Scanning electron microscopy imaging of the sample morphology indicates the film has grain size ranging from ∼100 to 500 nm (Fig. 1b), therefore, the regions of interest in Fig. 1a is not a single crystal but composed of multiple crystalline domains. The optical quality of the perovskite thin film was verified by recording the absorption and PL spectra of a ∼500 nm × 500 nm sample area (Fig. 1c, lower panel). The absorption and PL spectra from this localized area correlate well with those of bulk CH_3_NH_3_PbI_3_^15^, indicating that this region is indeed composed of high-quality perovskite crystallites.

To track charge-carrier dynamics in the perovskite film, we first identify transient absorption spectral signatures for excited carriers. The upper panel of Fig. 1c shows the ensemble transient absorption spectra of CH_3_NH_3_PbI_3_. There are two dominant photoinduced-bleaching bands at 480 and 760 nm. The photoinduced-bleaching band at ∼760 nm corresponds to the band-edge absorption^15^,^19^. Assignment of the 480-nm band and the broad photoinduced absorption is less obvious. A lower valence band to conduction band transition in a two-valence-band model was formerly proposed to explain the various excited-state spectral features^19^. The high-energy features have since also been attributed to an iodide-to-lead charge transfer transition that can account for both the 480-nm bleach and the broad photoinduced absorption signals^37^. Here we choose 580 nm as the probe wavelength for TAM measurements because it lies near the centre of the photoinduced absorption signal (away from any isosbestic point) and is free from the spectral shift due to the previously reported band filling effect at high pump intensities^15^.

The TAM image of the investigated region taken at 0-ps pump–probe delay is shown in Fig. 1d. In this measurement, the pump and probe beams overlap in space and the TAM image reflects the morphology of the CH_3_NH_3_PbI_3_ crystallite as can be seen from the correspondence between Fig. 1a,d. The pump–probe TAM signal in Fig. 1d is proportional to the initial density of excited carriers and has contribution from both electrons and holes. It is important to note that, unlike PL-based experiments, transient absorption is capable of monitoring both luminescent and non-luminescent species. This important distinction has been noted as a potential cause for the underestimation of diffusion length in PL studies of CH_3_NH_3_PbI_3_^12^,^26^,^38^.

### TAM imaging of carrier transport

**Figure 2:**
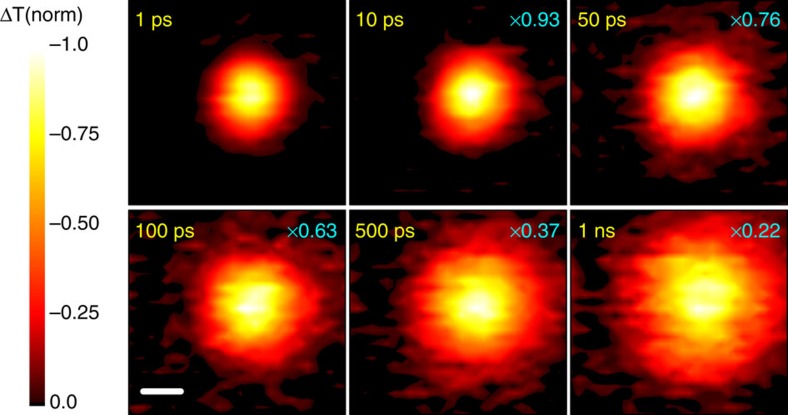
Two-dimensional transient absorption microscopy (TAM) imaging. The 2D imaging of the carrier density profile in CH_3_NH_3_PbI_3_ at different pump–probe delay times. The initial carrier density was 4 × 10^18^ cm^−3^ at zero delay time. For delay time >1 ps, the signal maximum in each image was normalized to the signal maximum at 1 ps. The scale bar in the figure is 300 nm.

**Figure 3:**
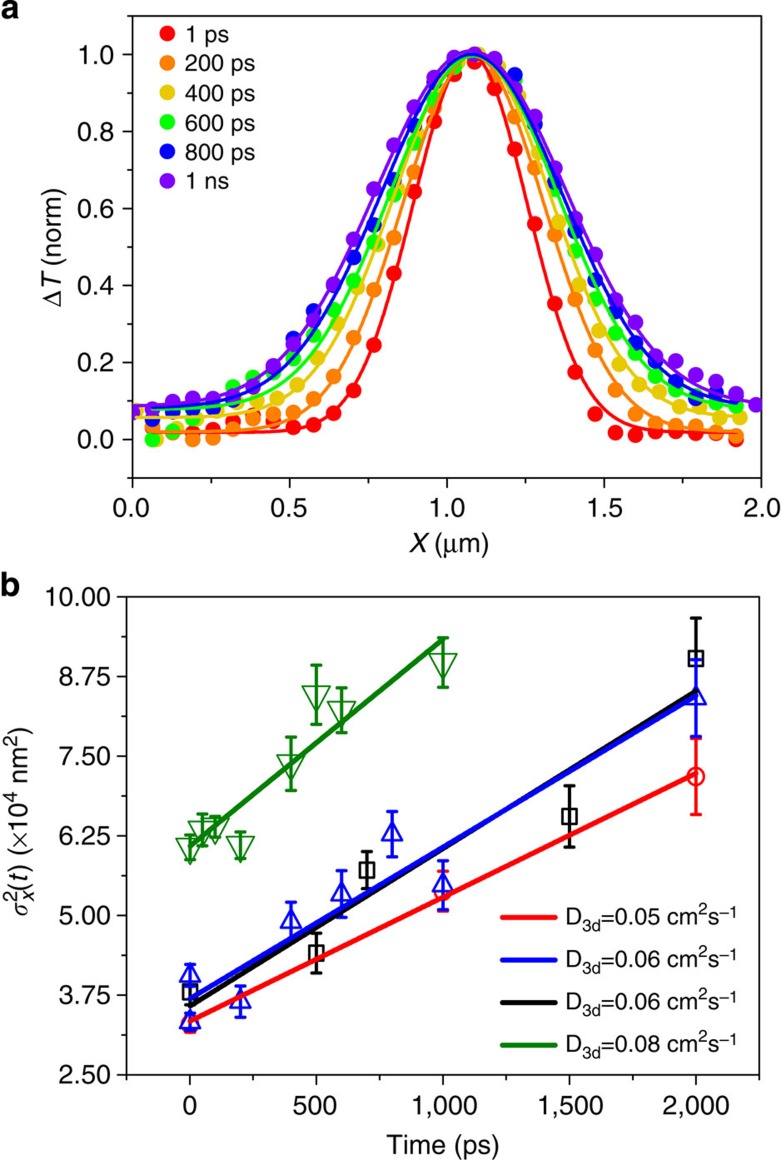
Diffusion coefficient estimated from time-dependent diffusion profiles. (**a**) Scans of excited-state density profile projected along one dimension. Data are acquired at different time delays using a pump density of 1 μJ cm^−2^ (initial carrier density of 2 × 10^17^ cm^−3^). The profiles are fitted by Gaussian functions. (**b**) Diffusion coefficients obtained through fitting the variances of Gaussian profiles, measurements have been performed on four different sample spots.

To image charge transport, a different modality of TAM is employed. The pump beam is fixed at a known position, while the probe beam is scanned relative to the pump position with a Galvanometer scanner to form an image (for more details, see Methods and Supplementary Fig. 2). In such TAM images, spatial distribution of carrier density as a function of pump–probe delay can be directly visualized. At 0 ps, the TAM image represents the initial photogenerated carrier population created by the pump pulse. At later delay times, the TAM image reflects carrier diffusion away from the initial excitation volume. Figure 2 presents TAM images of carrier density at different pump–probe delay times. Carrier diffusion is represented by the evolution of the carrier distribution profile as time elapses. Note that the precision in the carrier propagation distance is dictated by the smallest measurable change in the excited-state population profile and is determined by signal-to-noise^35^, rather than by the diffraction limit. By applying the megahertz frequency modulation on the pump beam^39^, shot-noise-limited detection is achieved with our TAM setup with change of transmission (Δ*T/T*) on the level of 5 × 10^−7^ detectable^40^. For the signal-to-noise levels shown in Fig. 2 and Fig. 3, this limit is ∼50 nm in resolving diffusion (see Supplementary Note 1 for more details on detection limit).

We note here that the sample region being investigated in Figs 2 and 3 is optically flat and homogeneous. As quantitative evidence, the histogram analysis of signal distribution in the TAM images with spatially overlapped pump and probe beams is well described by a Gaussian function and the variance is in good agreement with the noise level of the measurements (Supplementary Fig. 3). This implies that no further spatial normalization is required in analysing the diffusion imaging results.

### Modelling carrier transport in CH_3_NH_3_PbI_3_

The charge diffusion process in the perovskite films in absence of an electric field is modelled as in-plane two-dimensional (2D) diffusion because the signal is integrated over the out-of-plane direction. The population decay rate includes 2D diffusion from the excitation volume, relaxation to the ground state as well as higher order recombination process. We use the following phenomenological diffusion model,


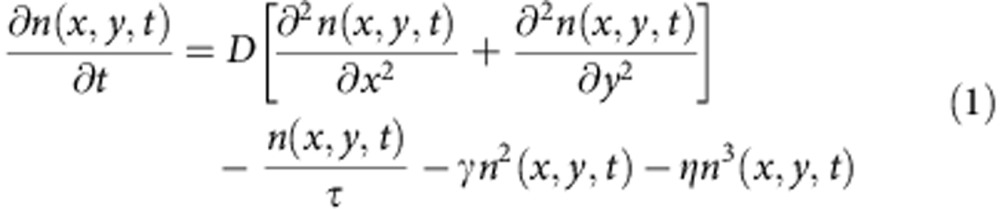


where *n*(*x,y,t*) is the carrier population as a function of position and time, *D* is the diffusion constant assuming isotropic diffusion, *τ* is the carrier lifetime, *γ* is the bimolecular recombination coefficient and *η* is the Auger recombination coefficient. Please note that *τ* includes both response of electrons and holes and we assume them to have similar lifetimes.

The initial population *n*(*x,y,0*) follows a 2D Gaussian distribution as created by a Gaussian pump beam at position (*x*_0_*, y*_0_) (the pump beam profile is shown in Supplementary Fig. 4):


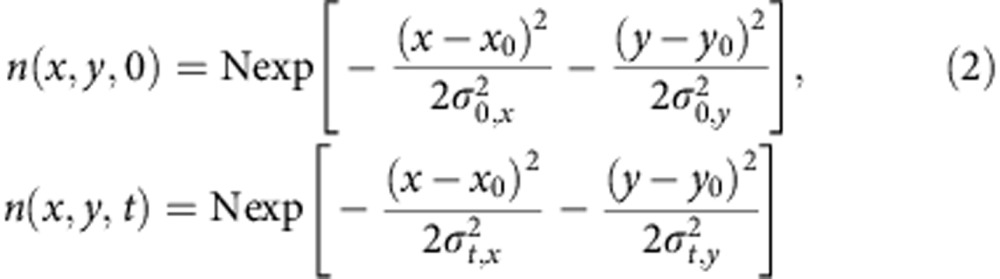


At low excitation intensity where the higher order recombination terms are negligible, the solution of equation (1) reduces into a 2D Gaussian distribution representing a spreading out population as a function of delay time *t*. In equation (2), 
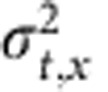
 and 
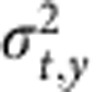
are the time-dependent variances of the Gaussian profiles along *x* and *y* direction. Carrier diffusion length *L* at delay time *t* is related to the variance of the excited-state density profile (*σ*^*2*^) by convolution rule (see Supplementary Note 2 for details). For instance, along the *x* axis,


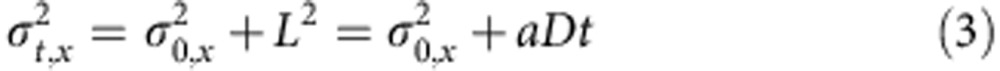


The coefficient *a* is related to the dimensionality of the diffusion process. For 2D diffusion, *a*=4. Therefore, the diffusion constant *D* is then given by


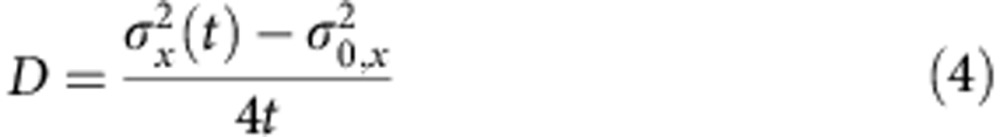


Using equation (4) and results from the TAM experiments we can directly determine the diffusion coefficient for charge carriers in CH_3_NH_3_PbI_3_.

To quantitatively model the excited-carrier diffusion, it is important to first perform the measurements at low fluence to minimize the influence of higher order recombination events and establish values for *D*. This is supported by previous reports that demonstrate the CH_3_NH_3_PbI_3_ excited-state decay proceeds primarily through first order processes at low carrier density^25^. Because no apparent angular anisotropy was observed in the 2D imaging results (see Fig. 2), we performed one dimensional (1D) scans to reduce data redundancy and allow longer time integration for improved signal-to-noise ratio.

As shown in Fig. 3a, the normalized 1D profiles of carrier density exhibit a time-dependent broadening. Those profiles are well described by Gaussian functions within the instrument noise level. Figure 3b plots the Gaussian variation as a function of pump–probe delay time for four different locations on the CH_3_NH_3_PbI_3_ thin film that are fitted to equation (3). The strong correlation between equation (3) and the experimental data suggests that carrier transport is diffusive in the low carrier density limit. The diffusion constant *D* measured at the four different locations ranges from 0.05 to 0.08 cm^2^ s^−1^.

**Figure 4:**
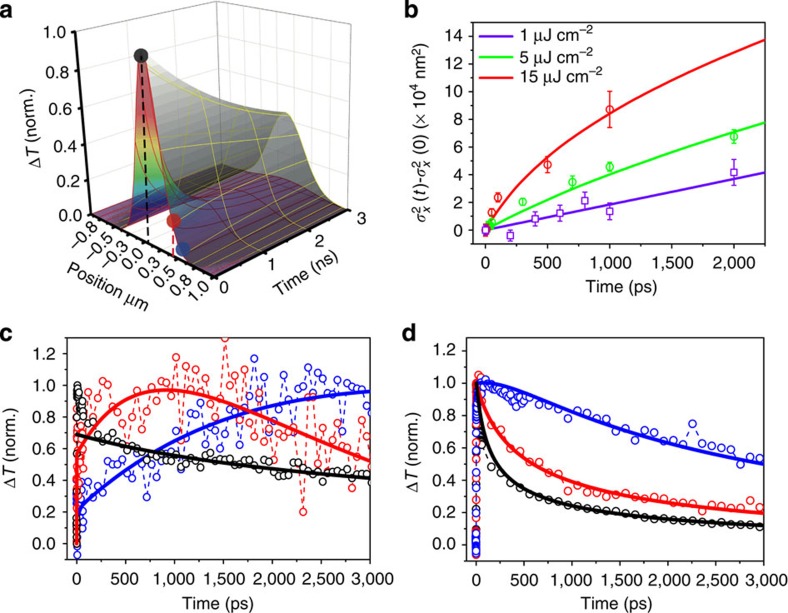
Numerical simulation/fitting of the experimental diffusion data. (**a**) The diffusion surface simulated by numerically solving equation (1) and then convolving the solution with probe Gaussian profile (∼230 nm FWHM). The initial condition is a gaussian source of ∼340 nm FWHM. Two pumping densities were simulated: ≤1 μJ cm^−2^ (carrier density 2 × 10^17^ cm^−3^, without bimolecular quenching) and 20 μJ cm^−2^ (4 × 10^18^ cm^−3^). Colour spots represent the probe beam position in the pump–probe offset scheme adopted in **c** and **d**. (**b**) 
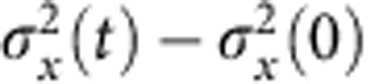
 changes as a function of delay time determined in TAM measurements using pump–probe offset scheme using 1 μJ cm^−2^ (2 × 10^17^ cm^−3^, purple), 5 μJ cm^−2^ (10^18^ cm^−3^, green) and 15 μJ cm^−2^ (3 × 10^18^ cm^−3^, red) pump fluence. The open symbols are experimental data, while the solid line are fits from the diffusion model as described by equation (1) (**c**,**d**) normalized excited-state density as a function of delay time probed at different pump–probe separations (black: 0 nm, red: 512 nm and blue: 768 nm). Two pump power density cases were studied: 2 μJ cm^−2^ (4 × 10^17^ cm^−3^) (**c**) and 20 μJ cm^−2^ (4 × 10^18^ cm^−3^) (**d**). The kinetic curves are fitted by a diffusion model as described by equation (1) and shown in solid lines. The only fitting parameter that is varied between **c** and **d** is the initial carrier density *n*(*x,y,*0).All curves have been normalized to their maxima.

To understand the spatial–temporal behaviour of charge transport, we modelled the carrier density as function of delay time and probe position using equation (1). The three-dimensional surface representing the temporal and spatial distribution of carriers is illustrated in Fig. 4a, which is simulated by numerically solving equation (1), taking into account convolution with the Gaussian profile of the probe beam (∼230 nm, full-width at half maximum).

We determine *τ* in our sample by fitting the kinetic traces to equation (1) under low carrier density. The result for an initial carrier density of 4 × 10^17^ cm^−3^ is shown in Fig. 4c. The diffusion model as described by equation (1) fits the dynamics taken at different pump–probe spatial separations very well as shown in Fig. 4c, which implies that the model is sufficient to capture the carrier transport processes. The only part that is not captured by the model is the short-time decay (<200 ps) when pump and probe beams are overlapped in space (Fig. 4c, black trace). This fast relaxation probably comes from other excited-state complexes do not diffuse because the transient absorption signal includes excited-state absorption of all species, and not just free electrons and holes. One possible candidate for such excited-state complex is the CH_3_NH_3_PbI–I_2_ charge transfer state^37^. *τ* obtained from the fittings shown Fig. 4c is 50±20 ns. This value is in good agreement with the PL lifetime measurement on the same sample, which gives a lifetime of 62 ns at a fluence of 50 nJ cm^−2^ (Supplementary Fig. 5). The intrinsic lifetime *τ* of excited carries in the CH_3_NH_3_PbI_3_ thin film depends on the defect concentration given specific fabrication conditions. Our results are consistent with the range of 4.5–140 ns reported for solution-processed polycrystalline films^16^,^19^,^41^.

Next, we investigate the effects of higher order recombination using pump intensity-dependent measurements of carrier diffusion. It is important to understand the role of higher order recombination in charge transport, that is, 
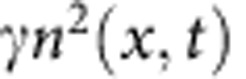
 and 
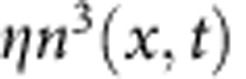
 in rate equation (1). as it defines the upper limit of carrier density in device operation^42^,^43^,^44^. Recent reports have suggested that CH_3_NH_3_PbI_3_ has a very low threshold for higher order effects such as bimolecular recombination and Auger processes^15^,^19^,^45^. We have explicitly taken carrier density in to account in our modelling by using equation (1) with the higher order recombination terms included. We have been able to satisfactorily model our measurements over more than one order of magnitude difference in carrier density using the same set of parameters. As shown in Fig. 4c,d, the carrier density distribution as a function of time and space is modelled at two initial carrier densities, 4 × 10^17^ cm^−3^ and 4 × 10^18^ cm^−3^. The only fitting parameter that is varied between Fig. 4c,d is the initial carrier density *n*(*x,y,*0). The resulting second and third order decay constants, are *γ*=6 × 10^−10^ cm^3^ s^−1^ and *η*=10^−27^ cm^6^ s^−1^, respectively. The second order decay constant correlates well with previous values determined from time-resolved terahertz spectroscopy of CH_3_NH_3_PbI_3_ thin films^25^.

The simulated results of equation (1) in Fig. 4 also show that the higher order recombination terms have profound effects on transport. As an illustration, 
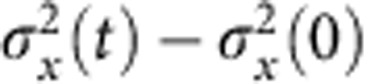
 as a function of time delay at different initial carrier densities (1 μJ cm^−2^ (2 × 10^−17^ cm^−3^), 5 μJ cm^−2^ (10^18^ cm^−3^) and 15 μJ cm^−2^ (3 × 10^18^ cm^−3^)) is plotted in Fig. 4b. It is clear that 
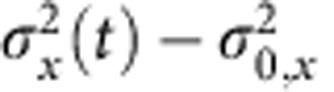
 grows linearly with delay time in the low carrier density limit. However, at high carrier densities, higher order terms make the broadening of the carrier diffusion artificially fast, suggesting that equations (2) and (3) are not valid at high-intensity excitation (>1 μJ cm^−2^) and the higher order recombination effects much be included to extract the right diffusion constants.

## Discussion

The *D* value measured in micron-sized regions of CH_3_NH_3_PbI_3_ is as high as 0.08 cm^2^ s^−1^, 2–8 times greater than previous estimates of 0.011–0.036 cm^2^ s^−1^ using PL-quenching measurements in bulk polycrystalline perovskite thin films^16^,^19^. This discrepancy most likely stems from unfavourable interactions at grain boundaries in bulk thin-film measurements. This is consistent with the very recent report on improving carrier mobility by enlarging grain size^46^. As photogenerated charges diffuse through the solution-processed semiconductor film, they are more likely to be scattered by defects leading to a shorter lifetime *τ* and smaller *D*. This is also reflected in our own results that reveal the measured diffusion length can vary depending on location of the CH_3_NH_3_PbI_3_ film (Fig. 3b). By extrapolating the TAM results over the full excited-state lifetime at low fluence (∼50 ns) and assuming a time-independent diffusion constant, we estimate a carrier diffusion length of ∼1.2 μm in polycrystalline CH_3_NH_3_PbI_3_.

We here compare our approach with other techniques. The charge diffusion in CH_3_NH_3_PbI_3_ polycrystalline films embedded in different architectures has been investigated using a variety of experimental techniques, including PL quenching, time domain terahertz and microwave photoconductance techniques^16^,^18^,^19^,^25^,^26^,^27^. The charge mobility in mesoporous Al_2_O_3_/CH_3_NH_3_PbI_3_ film and vapour-deposited CH_3_NH_3_PbI_3-*x*_Cl_*x*_ have been investigated by transient terahertz experiments^25^ on a similar time scale (nanoseconds) as this work, and has been determined to be on the same order but approximately 2.5–4 times higher than the values presented in this work. We attributed this discrepancy to two factors. First, the transient terahertz response reflects the sum of electrons and holes conductivities, while the transient absorption signal here gives an averaged value between the electrons and the holes depending on their absorption cross section at the probe wavelength. Second, sample fabrication details could also lead to different defect concentration that influences the carrier diffusivity. Solution processed Al_2_O_3_/ CH_3_NH_3_PbI_3_ mesoporous film and vapor-deposited CH_3_NH_3_PbI_3-*x*_Cl_*x*_ were used in ref. 25 and ref. 26, respectively, and we used solution-processed CH_3_NH_3_PbI_3_ film on glass. We note here that the carrier lifetime for our sample is 50–60 ns, which is shorter than some other reported values^41^. This could suggest a higher defect concentration in the film we measured, which could also lead to a smaller diffusion coefficient than what is reported in ref. 25. Transient microwave photoconductivity measurements^18^,^27^ have reported similar diffusion coefficient values (0.05–0.1 cm^2^ s^−1^) in neat CH_3_NH_3_PbI_3_ perovskite film^18^.

Our observations of spatial variation in carrier transport (Fig. 3b) underscore the importance of understanding the role of local morphology in dictating charge-carrier transport through organic–inorganic perovskites, and serve as a basis for further investigations into the relationship between structural order and diffusion in these materials. Very recently, measurements of carrier transport in large CH_3_NH_3_PbI_3_ perovskite single crystals revealed very long carrier lifetime and large diffusion length due to absence of grain boundaries and low defect concentration^28^,^29^,^46^. The diffusion coefficients at the single crystal level can be as high as 0.52–0.99 cm^2^ s^−1^, which sets an upper boundary for the mobility of free carriers generated in CH_3_NH_3_PbI_3_ perovskite structure.

It should be noted that even in the presence of grain boundaries, the diffusion coefficient only decrease by one order of magnitude compared with that in single crystal, much less of a decrease than other semiconductors such as silicon. Despite the inability to directly compare single crystal and thin-film results, grain boundaries and defect sites remain an important consideration in determination of transport properties in hybrid perovskites^47^,^48^. Another salient result from our experiments is the diffusive transport of charge carriers in CH_3_NH_3_PbI_3_ over several nanoseconds. This implies trap states do not significantly influence transport on this time scale since trapping and detrapping of carriers would lead to subdiffusive behaviour^35^. Trap states could become more important in limiting transport for the longer time scales. These transport properties support the exceptional functionality of polycrystalline organic–inorganic perovskites in optoelectronic device applications despite their low-energy processing requirements.

We interpret carrier transport in CH_3_NH_3_PbI_3_ using kinetic theory in semiconductors^49^, where carrier transport is limited by carrier scattering.


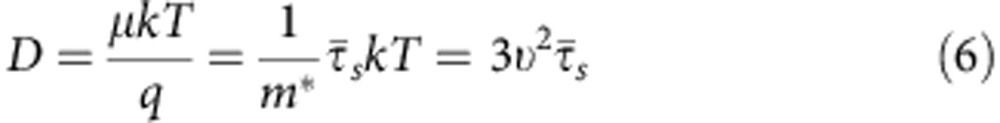


Here m* is the effective mass of the carriers, *μ* is the carrier mobility, *q* is the carrier charge, 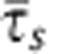
is the average scattering time of the carriers, and *ν* is root mean square velocity of the carrier, 
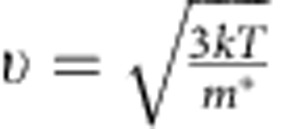
. Electronic structure calculations on the CH_3_NH_3_PbI_3_ lattice suggest that both electrons and holes have a small effective mass between 0.2 and 0.3 times the free electron rest mass, *m*_0_ (ref. 50).

At room temperature (300 K), 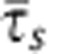 of CH_3_NH_3_PbI_3_ charge carriers is estimated to be ∼0.28 fs using a *D* value of 0.06 cm^2^ s^−1^ (average value from the four positions measured in our experiments), which is comparable to the scattering times in amorphous silicon (∼0.46 fs)^51^. The corresponding scattering length in the CH_3_NH_3_PbI_3_ structure is around 1 Å. 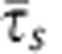
is in the subfemtosecond regime due to contributions from all possible scattering sources including phonons, defects and impurities, and is given by the sum of rates from all these processes^52^,^53^. The short scattering length implies that strong scattering occurs primarily within the lattice. Considering that the nearest equilibrium distance of an iodine atom and methylammonium group is only about 2.6 Å in CH_3_NH_3_PbI_3_, it is very possible that the organic ligand acts as the scattering source for the charges carried by iodine and lead^54^.

In summary, we present direct measurement of carrier transport in space and time by mapping carrier density in polycrystalline CH_3_NH_3_PbI_3_ thin films using transient absorption microscopy. Long-range carrier transport (∼220 nm in 2 ns) has been directly visualized for solution-processed CH_3_NH_3_PbI_3_ films. We successfully model spatial and temporal dependence of charge density using a diffusion model and show that higher order carrier recombination processes have profound impact on transport. Our measurements yield diffusion coefficient as high as 0.08 cm^2^ s^−1^ and an extrapolated diffusion length of ∼1.2 μm over a lifetime of 50 ns. These values are significantly larger than those inferred from bulk PL-quenching experiments in similar polycrystalline CH_3_NH_3_PbI_3_ thin films.

The spatially and temporally resolved measurements reported here establish an important step towards discerning the underlying transport properties of hybrid perovskite materials. Most notably, parameters relevant for device applications, such as diffusion length and the impact of higher order recombination on carrier transport have been elucidated directly, minimizing the added complexity and variability associated with bulk thin-film samples. This provides strong evidence that the morphology of the various perovskite films heavily influences carrier transport. Therefore, we posit that CH_3_NH_3_PbI_3_ can exhibit comparable photovoltaic performance relative to CH_3_NH_3_PbI_3-*x*_Cl_*x*_ in planar architectures provided the thin film is of sufficient quality. Direct determination of the transport properties in other hybrid perovskites using TAM will facilitate improved understanding of their properties. In addition, this technique can enable evaluation of the true photovoltaic potential of new materials without the variability associated with depositing contiguous, device-quality thin films.

## Methods

### Preparation of CH_3_NH_3_PbI_3_ perovskite film

Synthesis of methylammonium iodide (CH_3_NH_3_I) was carried out by dropwise addition of hydroiodic acid (57 wt%, aqueous, Alfa Aesar) into aqueous methylamine (40 wt%, Sigma Aldrich) under stirring at 0 °C for 2 h. The solvent was then removed by rotary evaporation and the crystals were washed in triplicate with diethyl ether and dried overnight under vacuum.

Equimolar quantities of PbI_2_ (99%, Acros Organics) and CH_3_NH_3_I were dissolved in *N*,*N*-dimethylformamide at 200 mg ml^−1^ to form the CH_3_NH_3_PbI_3_ precursor solution. The solution was heated to 70 °C and stirred for 1 h to ensure full solvation of the precursor components. Glass cover slips were cleaned by first sonicating in detergent solution followed by rinsing with deionized water and a second round of sonication in ethanol. The perovskite precursor solution was deposited on the clean glass substrates by spin coating at 2,000 r.p.m. for 30 s. The films were then treated at 100 °C for 20 min to form CH_3_NH_3_PbI_3_. All preparation steps were carried out in a dry nitrogen atmosphere.

### Optical measurements

The spun cast perovskite film was packaged by placing another microscope cover slip on top of the film forming a sandwich configuration and was sealed along the edges with epoxy to prevent any degradation due to air and moisture exposures. All the packaging procedures were carried out in the glove box filled with nitrogen atmosphere. Ultraviolet–visible absorption spectra were taken on a home-built absorption microscope system. The white light source (Tungsten Halogen Lamp, model 780, Newport Inc.) was coupled into fibre optics and focused on the perovskite film using a 100 × /NA(numerical aperture) 0.95 objective lens (Olympus UM Plan FI), which resulted in a focused beam with its diffraction full-width at half maximum <550 nm for the 400–850 nm spectral range. The transmitted light was detected by a charge-coupled device spectrometer (LR1, Aseq instruments). The reference spectra were taken on a bare area on the same substrate.

The setup used for transient absorption measurements of the bulk CH_3_NH_3_PbI_3_ perovskite sample has been described before^15^. For the TAM measurements, a schematic layout of the optical setup has been included in Supplementary Fig. 2 and is also described in previous publications^55^,^56^. Briefly, a Ti:Sapphire oscillator (Coherent Mira 900) pumped by a Verdi diode laser (Verdi V18) was used as the light source (output at 785 nm, 80 MHz repetition rate). 70% of the pulse energy was fed into the optical parametric oscillator (Coherent Mira OPO) to generate probe light at 580 nm, while the remainder 30% was doubled to 387 nm and serves as the pump beam. The repetition rate of both beams was reduced to 2.5 MHz using two clock-synchronized pulse pickers (Model 9200, Coherent Inc.). The pump beam was modulated at 1 MHz using an acoustic optical modulator (model R21080-1DM, Gooch&Housego). A 60 × /NA=1.49 objective (CFI Apo TIRF, Nikon Inc.) was used to focus the laser beams onto the sample, and the transmission light was then collected by another objective (60 × /NA=0.9) and detected by an avalanche photodiode (Hamamatsu C5331-04). For morphological TAM imaging, pump beam and probe beam were overlapped spatially and a piezo electric stage (P-527.3Cl, Physik Instrumente) was used to scan the sample to construct images such as Fig. 1b. In charge diffusion imaging as shown in Figs 2 and 3, the pump beam was fixed, while the probe beam was scanned by a pair of Galvanometer mirrors (Thorlabs Inc.). The sensitivity of TAM method utilized to extract the diffusion coefficient in the carrier diffusion process is determined not by optical diffraction limit but by signal-to-noise level of the TAM measurements. A detailed analysis can be found in the Supplementary Note 1.

## Additional information

**How to cite this article:** Guo, Z. *et al.* Spatial and temporal imaging of long-range charge transport in perovskite thin films by ultrafast microscopy. *Nat. Commun.* 6:7471 doi: 10.1038/ncomms8471 (2015).

## Supplementary Material

Supplementary Information Supplementary Figures 1-5, Supplementary Notes 1-2 and Supplementary References
