# Supplementary material for: Spatial and temporal imaging of long-range charge transport in perovskite thin films by ultrafast microscopy
Source: Nat Commun. 2015 Jun 23;6:7471. doi: 10.1038/ncomms8471 (PMC4557372; doi:10.1038/ncomms8471)
Supplement: Supplementary Information — Supplementary Figures 1-5, Supplementary Notes 1-2 and Supplementary References [file ncomms8471-s1.pdf]

Supplementary Figures

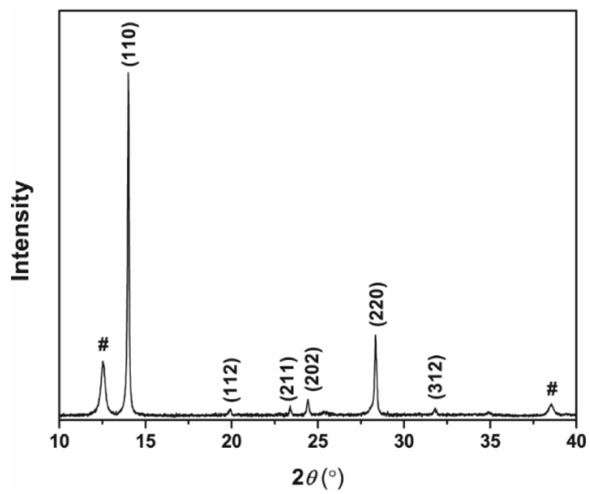

Supplementary Figure 1. X-ray diffraction pattern of $CH_3NH_3PbI_3$ film. Strong reflections of the (110) family of planes is characteristics of the preferred orientation of the perovskite crystallites. We were also able to detect signals from crystalline PbI_2 (denoted by #) in planar films annealed at 100 °C for 20 min. As reported previously[1], thermal annealing of polycrystalline perovskite films results in formation of a PbI_2 passivation layer primarily at grain boundaries.

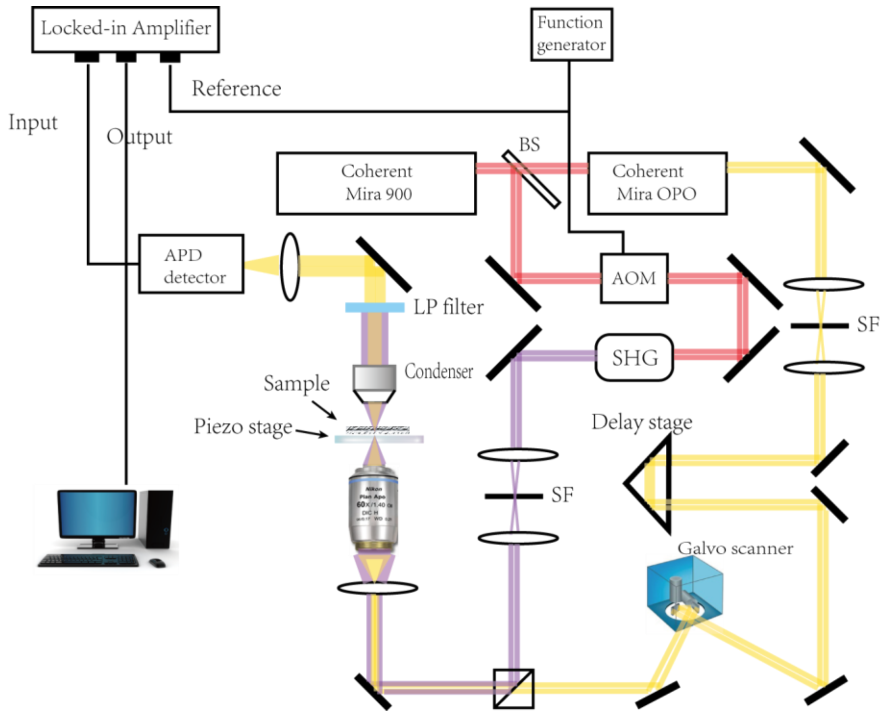

Supplementary Figure 2. Schematic layout of the TAM setup. Acronyms appear in the figure: OPO, optical parametric oscillator; BS, beam splitter; SF, spatial filter; AOM, acoustic optical modulator; SHG, second harmonic generation; LP filter, long-pass filter; APD detector, avalanche photodiode detector.

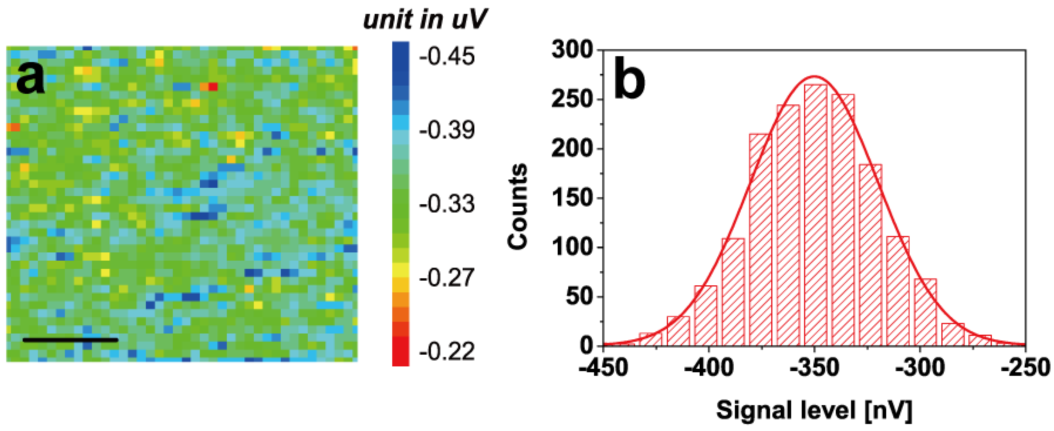

Supplementary Figure 3. **a.** TAM imaging of the sample region used for charge diffusion measurements at 0 ps. The scale bar is 500 nm. **b**. histogram of the pixel signals distribution. The distribution has been fitted with a gaussian function.

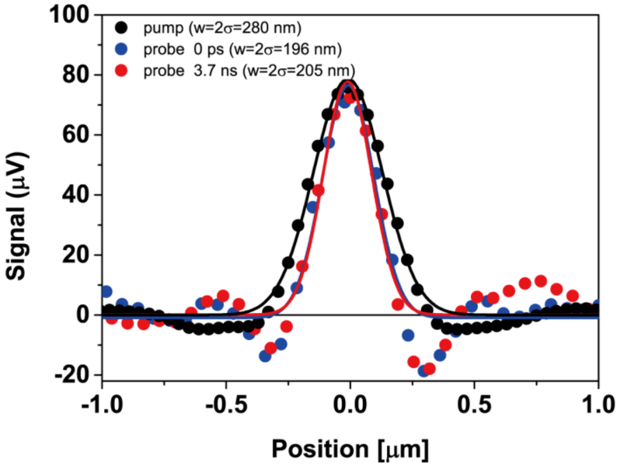

Supplementary Figure 4. Spatial resolution of the imaging system. In situ characterization of the imaging system's spatial resolution when two beams are in focus and give the signal maximum. The pump wavelength is at 400 nm and the probe wavelength is at 580 nm. The beam sizes are measured by scanning the pump/probe beams over a CdTe quantum wire that has a small cross-section (< 20 nm) using either a piezo electric stage (step size ~50 nm) or a galvo scanning mirror (step size ~64 nm).

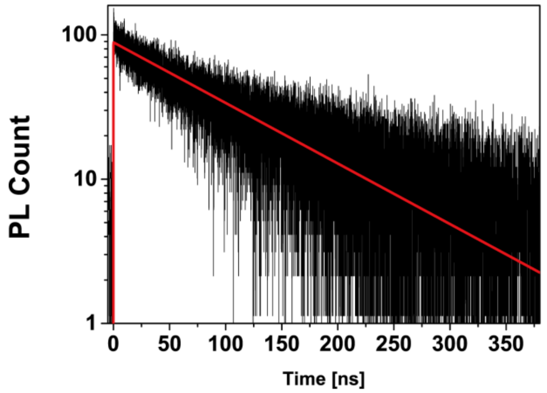

Supplementary Figure 5. Time resolved PL decay of $CH_3NH_3PbI_3$ perovskite film. Sample were excited using 470 nm pulse laser with a repetition rate of 4 MHz. The excitation fluence was set at 50 nJ/cm^2.

Supplementary Note 1

Factors that limits the resolution of TAM measurements

For the diffusion coefficient measurements, the critical resolution limit of TAM is limited by signal to noise rather than diffraction limit. From the equation used to obtain the diffusion coefficient (eq. 4 in the main text),

$$L^2 = \sigma_t^2 - \sigma_0^2 \quad (1)$$

One may perform a sensitivity analysis through differentiating the above expression and immediately find that

$$\Delta L = \sqrt{\frac{\sigma_t^2}{\sigma_t^2 - \sigma_0^2}\left(\Delta\sigma_t\right)^2 + \frac{\sigma_0^2}{\sigma_t^2 - \sigma_0^2}\left(\Delta\sigma_0\right)^2} = \sqrt{\Delta\sigma_t^2 + \left(\frac{\sigma_0}{L}\right)^2\left(\Delta\sigma_0^2 - \Delta\sigma_t^2\right)} \quad (2)$$

This shows that the error comes from the uncertainty of the Gaussian profiles measured at different time ($\Delta\sigma_t$ and $\Delta\sigma_0$), which is determined by the signal-to-noise of the detection system. A similar discussion on the resolution of such imaging approach can also be found in a prior work by Akselrod et al.[2], where a photoluminescence microscopy imaging technique was employed.

There are two mains sources of noise contributing to transient absorption experiments: laser fluctuation noise and electronic noise from the detection system (detector and lock-in amplifier, for instance). Noise due to laser intensity fluctuation can be effectively eliminated by using heterodyne lock-in detection with MHz modulation[3] where the intensity of the excitation beam (or additional local oscillator) is modulated by an acoustic-optical modulator. Subsequently, a lock-in amplifier referenced to this modulation frequency can sensitively extract the induced signal. The fluctuation of laser intensity (*1/f* noise) usually occurs at low frequency of dc to 10 kHz. When *f* is in the MHz range, the laser intensity noise becomes near the quantum shot noise limit, which is always present because of the Poisson distribution of the photon counts at the detector. The pixel dwell time should be significantly longer than the modulation period to allow for reliable demodulation for each pixel. Such a modulation scheme has been successfully applied to transient absorption microscopy[3].

Supplementary Note 2

Estimation of diffusion length

In the case where diffusion coefficient *D* is a constant, the diffusion equation is written as:

$$\frac{\partial n(\bar{r}, t)}{\partial t} = D \bullet \nabla^2 n(\bar{r}, t) \quad (1)$$

$n(\bar{r}, t)$ is the density distribution of the diffusing species. A general solution to this problem is $n(\bar{r}, t) = \frac{1}{(4\pi Dt)^{\frac{N}{2}}} \exp\left(-\frac{r^2}{4Dt}\right)$, *N* is the dimension being studied. *N*=1,2,3 corresponds to 1D, 2D and 3D diffusion respectively. The above solution has spherical symmetry and satisfies the normalization condition

$$\int_0^\infty n(\bar{r}, t) d\bar{r} = 1 \quad (2)$$

It can also be shown that the first moment and the second moment of the diffusing species' population density distribution has the following relationship in the spatial coordinates

$$\langle \bar{r}(t) \rangle = 0 \text{ and } \langle r^2(t) \rangle = \int_0^\infty n(\bar{r}, t) r^2 d\bar{r}. \quad (3)$$

In the TAM imaging case, the normalized diffusion pattern reflects a free, in-plane 2D diffusion process because the signal is integrated over the out-of-plane direction.

Therefore,

$$\langle r^2(t) \rangle = \int_0^\infty n(\bar{r}, t) r^2 d\bar{r} = \int_0^\infty n(\bar{r}, t) r^2 \bullet 2\pi r \bullet dr = 4Dt \quad (4)$$

For two time delays, t_1 and t_2, the variance of the ensemble distribution is given by

$$\langle r^2(t_1) \rangle = \sigma_1^2 = 4Dt_1, \text{ and } \langle r^2(t_2) \rangle = \sigma_2^2 = 4Dt_2 \quad (5)$$

$D = \frac{\sigma_1^2 - \sigma_2^2}{4\Delta t_{12}}$, if we take $t_1=\tau$ and $t_2=0$, then $D = \frac{\sigma^2(\tau) - \sigma_0^2}{4\tau}$. The diffusion length is defined over an average lifetime τ, $L = \sqrt{4D\tau}$, and $L^2 = \sigma^2(\tau) - \sigma_0^2$

Supplementary References

1 Chen, Q. *et al.* Controllable self-induced passivation of hybrid lead iodide perovskites toward high performance solar cells. *Nano Lett.* **14**, 4158-4163, (2014).

2 Akselrod, G. M. *et al.* Visualization of exciton transport in ordered and disordered molecular solids. *Nat. Commun.* **5**, 3646, (2014).

3 Chong, S., Min, W. & Xie, X. S. Ground-State Depletion Microscopy: Detection Sensitivity of Single-Molecule Optical Absorption at Room Temperature. *J. Phys. Chem. Lett.* **1**, 3316-3322, (2010).
